# Investigating metabolic dysregulation in serum of triple transgenic Alzheimer’s disease male mice: implications for pathogenesis and potential biomarkers

**DOI:** 10.1007/s00726-023-03375-1

**Published:** 2024-02-05

**Authors:** Hongbin Zhuang, Xueshan Cao, Xiaoxiao Tang, Yongdong Zou, Hongbo Yang, Zhiyuan Liang, Xi Yan, Xiaolu Chen, Xingui Feng, Liming Shen

**Affiliations:** 1 College of Life Science and Oceanography, Shenzhen University, Shenzhen, 518071 People’s Republic of China; 2 Shenzhen-Hong Kong Institute of Brain Science-Shenzhen Fundamental Research Institutions, Shenzhen, 518055 People’s Republic of China; 3 Center for Instrumental Analysis, Shenzhen University, Shenzhen, 518071 People’s Republic of China; 4 The Key Laboratory of Environmental Pollution Monitoring and Disease Control, Ministry of Education, School of Public Health, Guizhou Medical University, Guiyang, 550025 People’s Republic of China

**Keywords:** Alzheimer’s disease, Biomarker, Serum, Target metabolomics, Triple-transgenic AD mice

## Abstract

Alzheimer’s disease (AD) is a multifactorial neurodegenerative disease that lacks convenient and accessible peripheral blood diagnostic markers and effective drugs. Metabolic dysfunction is one of AD risk factors, which leaded to alterations of various metabolites in the body. Pathological changes of the brain can be reflected in blood metabolites that are expected to explain the disease mechanisms or be candidate biomarkers. The aim of this study was to investigate the changes of targeted metabolites within peripheral blood of AD mouse model, with the purpose of exploring the disease mechanism and potential biomarkers. Targeted metabolomics was used to quantify 256 metabolites in serum of triple transgenic AD (3 × Tg-AD) male mice. Compared with controls, 49 differential metabolites represented dysregulation in purine, pyrimidine, tryptophan, cysteine and methionine and glycerophospholipid metabolism. Among them, adenosine, serotonin, N-acetyl-5-hydroxytryptamine, and acetylcholine play a key role in regulating neural transmitter network. The alteration of S-adenosine-l-homocysteine, S-adenosine-l-methionine, and trimethylamine-N-oxide in AD mice serum can served as indicator of AD risk. The results revealed the changes of metabolites in serum, suggesting that metabolic dysregulation in periphery in AD mice may be related to the disturbances in neuroinhibition, the serotonergic system, sleep function, the cholinergic system, and the gut microbiota. This study provides novel insights into the dysregulation of several key metabolites and metabolic pathways in AD, presenting potential avenues for future research and the development of peripheral biomarkers.

**Supplementary Information:**

The online version contains supplementary material available at 10.1007/s00726-023-03375-1.

## Introduction

Alzheimer’s disease (AD) is a neurodegenerative disease that is a serious health risk for the elderly (Niedowicz et al. 2014; Lang et al. 2012). It is characterized clinically by memory impairment, aphasia, apraxia, agnosia, impairment of visuospatial skills, executive dysfunction, and personality and behavioral changes, and the etiology is still unknown (Conejero-Goldberg et al. 2008). The main pathological features are senile plaque formed by β-amyloid (Aβ) deposits and neurogenic fibrillary tangles (NFT) formed by abnormal aggregation of tau proteins (Abe et al. 2020). The critical factors of AD pathogenesis included genetics, aging, environmental factor, lifestyle habits, viral and bacterial infections, sleep, and the potential role of the microbiota (Guo et al. 2020). The presence of Aβ, tau proteins, as well as glia significantly contribute to the pathogenesis of AD. These factors can disrupt synaptic plasticity, impair synaptic function, disturb intracellular Ca^2+^ homeostasis, trigger inflammatory-immune responses, induce mitochondrial damage, generate oxidative stress, and cause abnormal lipid and energy metabolism (Pang et al. 2022; Parodi-Rullán et al. 2021; Yu et al. 2009; Forman et al. 2005; Shen et al. 2016). Ultimately, the cumulative effects of these processes result in neuronal apoptosis and dysregulation, and the manifestation of disease. Because of its complex etiology, long incubation period and slow progress, the disease has a 15–20-year evolution process (Diniz-Filho et al. 2017). Early detection and intervention are the main measures in the management of AD. Before the lesion is too serious to be cured, it is essential to make early diagnosis of AD and intervene with medication (Gurevich et al. 2017; Yu et al. 2021; Calvini et al. 2009).

At present, clinical diagnostic methods for AD include psychiatric cognitive scale tests, neuroimaging, and biomarker tests (Shin et al. 2021; Silvestro et al. 2019). However, diagnoses on scales are subjective, and early imaging is focused on positron emission tomography (PET), which is expensive (Lim et al. 2019; Park et al. 2019). Biomarker testing based on cerebrospinal fluid (CSF), and collection requires lumbar puncture is not conducive to screen biomarker (Shin et al. 2021; Zhao et al. 2021a). Blood-based biomarker research has become a current hotspot but it has limited clinical application (Cheng et al. 2020; Shen et al. 2019). The main reason is that the levels of commonly used blood diagnostic markers, such as those associated with Aβ and tau, are extremely low, and existing methods are still unable to meet the requirements of routine detection in terms of sensitivity or detection cost (Mielke et al. 2017; Shen et al. 2019). Additionally, since the pathogenesis of AD is multidimensional and has different pathological characteristics, which is also considered as a metabolic network failure disease (Kaddurah-Daouk et al. 2013; Kang et al. 2017; Toledo et al. 2017). Serum metabolites are expected to distinguish healthy controls and moderate cognitive impairment groups (Zhang et al. 2021b; Toledo et al. 2017) or AD groups (Huo et al. 2020). Therefore, there is an urgent need to conduct biomarker research from different perspectives, explore novel specific early diagnostic markers, and establish highly sensitive and cost-controllable detection methods to achieve early detection and intervention (Shen et al. 2019). Moreover, the specific treatment for AD remains difficult to achieve. Consequently, it is also an urgent need to further clarify the mechanism and search for effective new drug targets.

High-throughput omics approaches offer technical advantages in exploring disease mechanism. Metabolomics provides a powerful and potential method for finding diagnostic markers and new therapeutic targets for AD. In recent years, it has been applied to the research of AD (Xie et al. 2021; Hao et al. 2018; Varma et al. 2018; Yin et al. 2023). As expected, an increasing number of blood metabolites in AD have been successfully identified that are associated with inflammation, oxidative stress, mitochondrial dysfunction, and neuronal damage (Snyder et al. 2014; González-Domínguez et al. 2014; Jia et al. 2022). Interestingly, Aβ was found to be associated with different peripheral metabolites such as bile acid, uric acid, homocysteine, cholesterol and cortisol (Nho et al. 2019; Kiddle et al. 2012; Oxenkrug et al. 2017; Hu et al. 2020; Sun et al. 2020). Abnormalities in serum phosphatidylcholine may reflect disruption of brain membrane integrity (Nho et al. 2021). Lower serum serotonin levels are associated with cognitive decline in AD patients, while higher levels of triacyl carnitine indicate metabolism with long-chain fatty acids for energy supply (Whiley et al. 2021; Huo et al. 2020). In addition, 11 metabolites involved in different metabolic pathways were identified as biomarkers that can distinguished AD from control groups (Sun et al. 2020). A study from 11 cohorts identified subcomponents of high-density lipoprotein (HDL), docosahexaenoic acid (DHA), ornithine, glutamine, and glycoprotein acetyl as biomarkers (van der Lee et al. 2018). Moreover, several metabolites including arachidonic acid, N, N-dimethylglycine, thymine, glutamine, glutamic acid, and cytidine were identified to distinguish AD patients from normal controls (Wang et al. 2014). Therefore, these findings support that those changes in peripheral blood metabolites can indeed reflect the pathophysiological mechanism of AD, and these metabolites have the potential to be served as diagnostic markers. However, more studies are needed to address the specificity and repeatability of metabolic biomarkers, as well as more in-depth studies from a metabolomic perspective.

Targeted metabolomics has significant quantification advantages over non-targeted metabolomics (Zhou and Yin 2016; Lin et al. 2022). In contrast to single detection of a metabolite, a widely targeted metabolic profile in the blood can accurately reflect interrelated biochemical pathway perturbations and identify more effective biomarkers (Venkataraman et al. 2022; Leyane et al. 2022). For example, a targeted metabolomic study showed that three serum acylcarnitines negatively predicted the risk of AD events and cognitive decline from antemortem blood and postmortem brain (Huo et al. 2020). The triple transgenic AD mouse model (3 × Tg-AD) showed the simultaneous existence of Aβ plaques and NFT formation, which mimics the characteristic pathology of human AD, and is an ideal and commonly used mouse model for AD research (Bai et al. 2020; Oddo et al. 2003a; Kim et al. 2018). The aged mice treated with plasma from young wild-type mouse exhibited an improvement of amyloid pathology in brain indicating an interplay between the brain and blood (Zhao et al. 2020). The study of metabolites in peripheral blood can provide clues to explain changes in the brain. In our previous study, we conducted a targeted metabolomic study on 200 metabolites in their hippocampus and eyes (Shen et al. 2023). The results showed significant differences in metabolites between 6-month-old 3 × Tg-AD mice and control mice (Zhao et al. 2021b). In this study, the number of target metabolites was increased to 530. We performed a targeted metabolomics in serum from 6-month-old 3 × Tg-AD mice and control group and aimed to explore the underlying mechanism and potential biomarkers. Moreover, the dysregulated metabolites can contribute some information for the diagnosis and treatment of AD.

## Materials and method

### Animal and serum samples collection

The original breeding pairs of 3 × Tg-AD mice (Oddo et al. 2003b) harboring human PS1M146V, human APPswe, and human tauP301L and wild-type (WT) control mice (B6:129SF2/J) were purchased from Jackson Laboratories (Bar Harbor, Maine, USA). The mice used in experiment were born in our laboratory. Both 3 × Tg-AD and WT mice were maintained in controlled conditions (12 h light/12 h dark cycle, temperature 22 °C, humidity 50–60%, fresh food, and water ad libitum). In this study, 6-month-old WT male mice (*n* = 8) and 6-month-old AD male mice (*n* = 8) were assigned to WT and 3 × Tg-AD group, respectively. All protocols were strictly observed for animal care and welfare according to the NIH guidelines (NIH publication No. 85–23, revised 1985). The study was approved by the Animal Ethical and Welfare Committee of Shenzhen University (Permit Number: AEWC-20140615-002).

At the age of 6 months, the mice were anesthetized with isoflurane (3% initial dose, 1.5% maintenance dose). Blood samples were collected from anesthetized animals by cardiac puncture within disposable sterilized syringe at 9:00 a.m. Preoperative blood (0.6–1.0 mL) was collected in the Eppendorf tube without additives through cardiac puncture. The blood was coagulated at room temperature for 60 min and then centrifuged at 3600 × *g* for 10 min. The supernatant, serum, was transferred to new Eppendorf tubes and centrifuged at 6500 × *g* for 3 min. The serum sample (100–200 µL) was stored at  − 80 °C until use.

### Metabolites’ extraction from serum

The 100 μL of serum sample was diluted by 100 μL ddH_2_O and mingled with ice-cold extraction buffer (800 μL, methanol/acetonitrile, 1/1, v/v, containing internal standard (IS)) (Table S1). After vortex, the sample was sonicated for 5 min in ice-water bath and incubated in  − 40 °C for 2 h. Then, the sample was centrifugated at 12,000 rpm for 15 min at 4 ℃. The 800 μL supernatant was transferred into fresh tube and dried in a vacuum concentrator. The dried samples were reconstituted in 60% acetonitrile by sonication on ice for 10 min. The reconstituted samples were centrifugated at 12,000 rpm for 15 min at 4 ℃ and supernatant was transferred to a fresh glass vial for liquid chromatography-mass spectrometry (LC/MS) analysis.

### Targeted LC–MS analysis

Quality control (QC) samples were collected from a pooled sample of all serum samples and used for data normalization. Blank samples (75% ACN in water) and QC samples were injected every eight samples during acquisition. The highest concentration of IS mixture is set to S1, S1/2 for the next level, and dilute step by step to make the standard curve. When the relative standard deviation of IS response is less than 15%, the system is considered stable. The internal standard method was used to quantify targeted metabolites. The ratio of the standard and IS was utilized to rectify instrument fluctuation. The establishment of a database containing specific ions of targeted metabolites was accomplished through the standards and the Human Metabolome Database (HMDB, https://hmdb.ca). The chromatography separation was carried out using a H-Class (Waters, USA) ultra-high-performance liquid chromatography, equipped with a Waters Atlantis Premier BEH Z-HILIC Column (1.7 µm, 2.1 × 150 mm). The flow rate was 0.5 mL/min, and the sample injection volume was 6 μL. The mobile phase consisted of 10 mM ammonium acetate in 20% acetonitrile (8:2 = water: acetonitrile) (pH = 9) (A) and 90% acetonitrile (9:1 = acetonitrile: water) (B) (pH = 9). The linear gradient was set as follows: 0–1 min: 1% B, 1–8 min: 1% B to 100% B, 8–10 min: 100% B, 10–10.1 min: 100% B to 1% B, 10.1–12 min: 1% B. The column temperature was 25 ℃. The auto-sampler temperature was 8 ℃. A SCIEX 6500 triple quadrupole mass spectrometer (AB SCIEX, USA) equipped with IonDrive Turbo V ESI ion source was applied for assay development. Curtain Gas = 35 psi, IonSpray Voltage =  + 5000 V/−4500 V, Temperature = 400 °C, Ion Source Gas 1 = 50 psi, Ion Source Gas 2 = 50 psi. The scan type was selected as multiple response monitoring (MRM) to detect the specific parent ion and daughter ion in samples and identify them by database. Duration = 22 min, delay time = 0, cycles = 2640, cycle = 0.5 s.

### Quantitative data process

The raw data consisted of 2 quality control and 16 experimental samples, with total detection of 530 targeted metabolites (Table S2). The MS raw data files were converted to the mzXML format by ProteoWizard (RRID: SCR_012056). Subsequently, data were processed by R package XCMS (version 3.2) (RRID: SCR_015538) to generate a data matrix that consisted of the retention time (RT), mass-to-charge ratio (*m*/*z*) values, and peak intensity. Metabolic features detected less than 80% in all the QC samples were discarded. The missing values in the original data were simulated, which was the half minimum method to fill. The final concentration (*C*_F_, μmol/L) of sample was calculated by means of calculated concentration (*C*_C_, μmol/L) multiplied by dilution factor (Dil). The targeted metabolite concentration (*C*_M_, nmol/L) was equal to the *C*_F_ multiplied by the final volume (*V*_F_) and the concentration coefficient in the process of pretreatment, divided by the sampling volume *V* (nmol/L).

### Metabolomics data analysis

The metabolomics data analysis was conducted with R (https://www.r-project.org) (RRID: SCR_001905) and online versions of MetaboAnalyst (http://www.metaboanalyst.ca) (RRID: SCR_015539) as described previously (Zhao et al. 2021b; Zhang et al. 2021a). Orthogonal partial least-squares discrimination analysis (OPLS-DA), permutation test, and principal component analysis (PCA) were performed using the SIMCA software (v14.1, Sartorius Stedim Data Analytics AB, Umea, Sweden) (RRID: SCR_014688). The differential expression metabolites (DEMs) were conducted using the R package Limma (v3.48.3) (RRID:SCR_010943) (Ritchie et al. 2015) and variable importance in projection (VIP) scores from OPLS-DA analysis. The cut off values of 1.2-fold change (FC) for up-regulated and down-regulated, *p*-value < 0.05 and VIP > 1 were set for significantly differential metabolites in two groups after Benjamin–Hochberg correct. The differential metabolites were selected as candidate to perform enrichment analysis. Kyoto Encyclopedia of Genes and Genomes (KEGG) enrichment analysis were performed in MetaboAnalyst and the pathways were considered significantly in line with impact > 0 and *p* < 0.05 after Benjamin–Hochberg correct. Metabolite set enrichment analysis (MSEA) based on KEGG and The Small Molecule Pathway Database (SMPDB) were performed using the online tool MetaboAnalyst and the pathways were significant in line with *p* < 0.05. The heatmaps were drawn using the R package pheatmap (v1.0.12) (RRID:SCR_016418). The order statistics was calculated in the R package robust ranked aggregation (v1.1) (Kolde et al. 2012) and the pathways were significant in line with score < 0.05. The Spearman’s two matrix correlation analysis was estimated in the R package psych (v2.2.3) (RRID: SCR_021744) after Benjamin–Hochberg correct (*p* < 0.05). The graphical visualization tool was ggplot2 (v3.3.5) (RRID:SCR_014601).

## Results

### Metabolic profiling to distinguish the 3 × Tg-AD and WT mice serum

By detecting 530 metabolites from 3 × Tg-AD and WT mice serum, a total of 256 targeted metabolites were identified and quantified (Table S3). They were mainly involved in aminoacyl-tRNA biosynthesis, arginine biosynthesis, glycine, serine and threonine metabolism, alanine, aspartate and glutamate metabolism, purine metabolism, pyrimidine metabolism, pantothenate and CoA biosynthesis, tryptophan metabolism, cysteine and methionine metabolism, glycerophospholipid metabolism, and arginine and proline metabolism (Fig. S1).

**Fig. 1:**
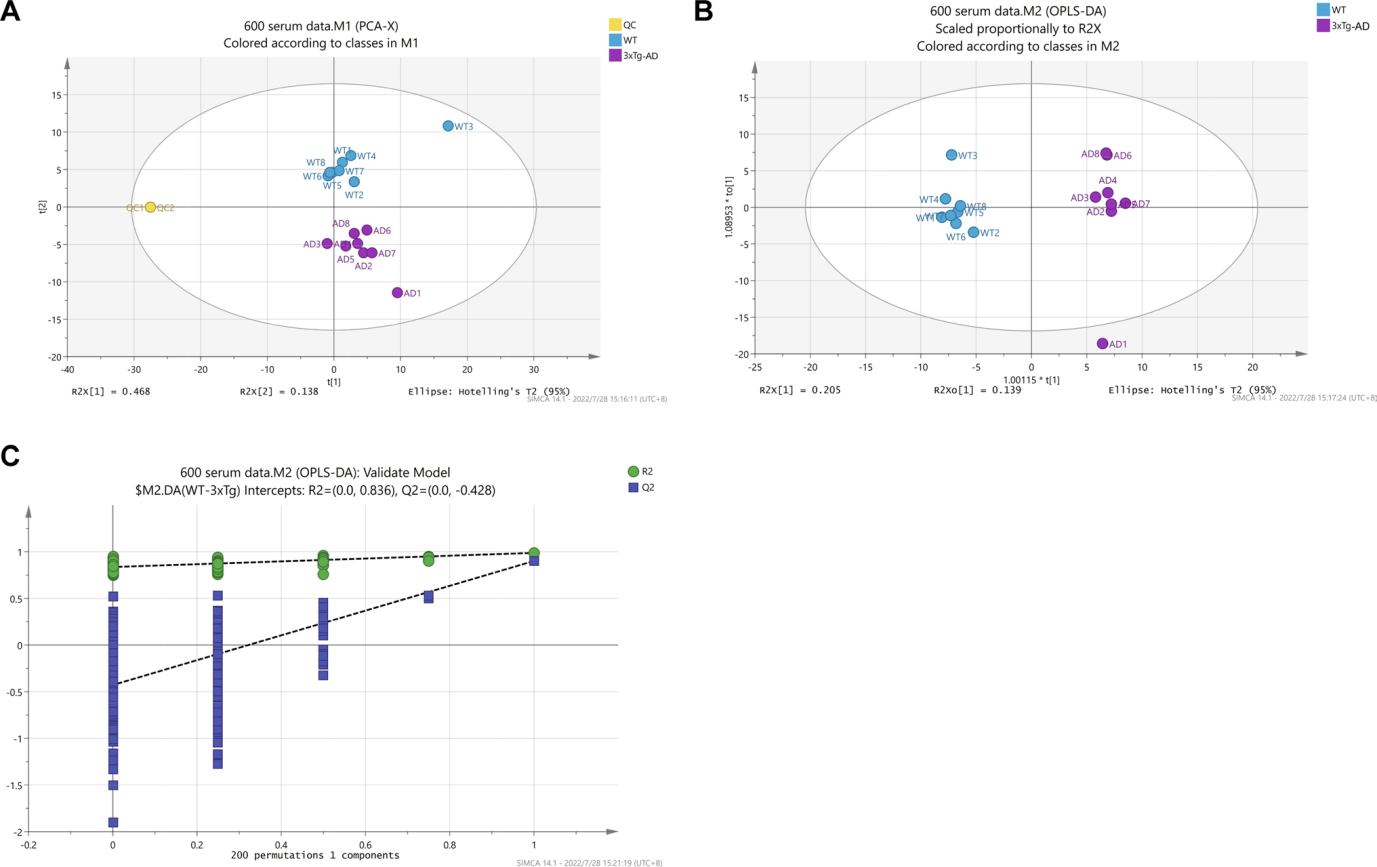
PCA and OPLS-DA analysis of metabolites in this study. **a** PCA score plot of metabolites in 3 × Tg-AD and WT mice. **b** OPLS-DA scatter plot of 3 × Tg-AD and WT mice. **c** The results of OPLS-DA model replacement test in 3 × Tg-AD and WT mice

Multivariate statistical analyses were used to assess separation between groups. The results of PCA showed significant differences between 3 × Tg-AD and WT mice groups (Fig. 1a). The contribution of variables was characterized using the supervised OPLS-DA model, which showed that there were significant differences between metabolites of 3 × Tg-AD and WT mice (Fig. 1b). The model was validated by 200 times permutation test, which showed low *Q*^2^ values and a negative intercept of the regression line on the *y*-axis, indicating that the model was robust and not over-fitted (Fig. 1c). The cluster analysis of 256 targeted metabolites showed the most of 3 × Tg-AD mice could be distinguished in one cluster (Fig. S2).

### Identification of differential metabolites in serum

**Fig. 2:**
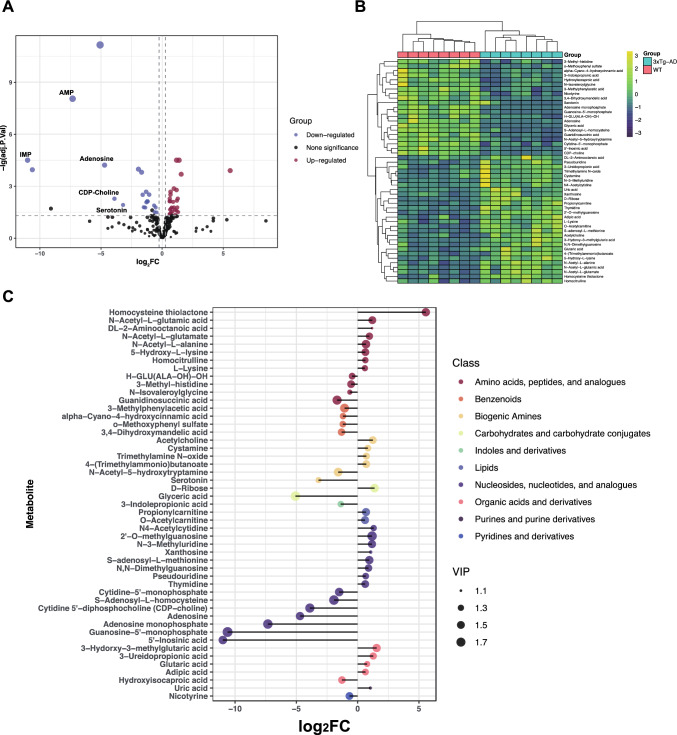
Overview of differential expression metabolites. **a** The volcano analysis of DEMs. **b** The stick plot shows the VIP value, classification, and log foldchange of DEMs. **c** The heatmap display 49 DEMs between 3 × Tg-AD and WT mice

Volcano plot analysis revealed that a total of 49 differential expression metabolites (DEMs) were identified between the 2 groups. Compared with WT mice, 28 metabolites were up-regulated, while 21 metabolites were down-regulated in the serum of AD mice (Fig. 2a, Table 1 and Table S4). These 49 DEMs were used for cluster analysis, and they were able to effectively distinguish AD from WT mice (Fig. 2b). The matchstick plot shows the classification categories of DEMs, which were mainly involved in amino acids, peptides and analogues, nucleosides, nucleotides and analogues, benzenoids, biogenic amines, and organic acids and derivatives (Fig. 2c).

**Table 1:** The serum differential metabolites between 6-month-old 3 × Tg-AD mice and WT mice

Compound name	HMDB ID	log_2_FC	Foldchange	Adj. *P* Val	VIP
Homocysteine thiolactone	HMDB0002287	5.56	47.17	1.26E-04	1.57
3-Hydorxy-3-methylglutaric acid		1.55	2.93	2.04E-04	1.56
d-Ribose	HMDB0000283	1.38	2.60	3.15E-05	1.62
N4-Acetylcytidine	HMDB0005923	1.31	2.47	3.12E-02	1.30
3-Ureidopropionate (3-Ureidopropionc acid)	HMDB0000026	1.27	2.41	5.21E-03	1.42
Acetylcholine	HMDB0000895	1.24	2.36	1.68E-02	1.49
N-Acetyl-l-glutamic acid	HMDB0001138	1.20	2.29	6.79E-03	1.50
dl-2-Aminooctanoic acid	HMDB0000991	1.18	2.26	2.62E-02	1.10
2′-O-methylguanosine		1.17	2.26	3.15E-05	1.77
N-3-Methyluridine		1.17	2.25	1.66E-03	1.55
Xanthosine	HMDB0000299	1.07	2.09	4.41E-02	1.11
Uric acid		1.04	2.05	2.30E-02	1.11
N-Acetyl-l-glutamate		0.96	1.94	1.68E-02	1.42
S-adenosyl-l-methionine (SAM)		0.95	1.94	1.37E-03	1.58
N,N-Dimethylguanosine	HMDB0004824	0.89	1.85	8.04E-03	1.44
Cystamine	HMDB0250701	0.83	1.78	2.99E-02	1.35
Glutaric acid	HMDB0000661	0.78	1.71	1.70E-02	1.28
Trimethylamine N-oxide (TMAO)		0.71	1.64	3.43E-02	1.38
4-(Trimethylammonio)butanoate	HMDB0001161	0.70	1.63	1.99E-02	1.50
Propionylcarnitine	HMDB0000824	0.68	1.60	2.10E-03	1.55
N-Acetyl-l-alanine	HMDB0000766	0.68	1.60	3.06E-03	1.61
Pseudouridine	HMDB0000767	0.66	1.58	4.00E-02	1.34
Adipic acid	HMDB0000448	0.64	1.55	2.27E-02	1.36
5-Hydroxy-l-lysine		0.63	1.55	7.45E-03	1.49
Homocitrulline	HMDB0000679	0.63	1.55	4.00E-02	1.31
Thymidine	HMDB0000273	0.62	1.53	5.21E-03	1.49
O-Acetylcarnitine		0.60	1.52	7.45E-03	1.52
l-Lysine	HMDB0000182	0.59	1.50	4.12E-02	1.30
H-GLU(ALA-OH)-OH		− 0.45	0.73	3.26E-02	1.36
3-Methyl-histidine		− 0.54	0.69	1.70E-02	1.49
N-Isovaleroylglycine	HMDB0000678	− 0.64	0.64	3.08E-02	1.20
Nicotyrine	HMDB0255591	− 0.67	0.63	1.30E-02	1.49
3-Methylphenylacetic acid	HMDB0002222	− 1.07	0.48	3.06E-03	1.68
alpha-Cyano-4-hydroxycinnamic acid	HMDB0248196	− 1.19	0.44	7.58E-03	1.32
o-Methoxyphenyl sulfate	HMDB0060013	− 1.21	0.43	7.45E-03	1.34
Hydroxyisocaproic acid	HMDB0000746	− 1.28	0.41	8.48E-03	1.46
3,4-Dihydroxymandelic acid	HMDB0001866	− 1.31	0.40	2.10E-03	1.45
3-Indolepropionic acid	HMDB0002302	− 1.38	0.38	1.73E-02	1.32
Cytidine-5′-monophosphate (CMP)	HMDB0000095	− 1.50	0.35	1.64E-02	1.61
N-Acetyl-5-hydroxytryptamine (N-Acetyl-5-HT)	HMDB0001238	− 1.59	0.33	3.13E-03	1.64
Guanidinosuccinic acid	HMDB0003157	− 1.68	0.31	1.56E-04	1.72
S-Adenosyl-l-homocysteine (SAH)	HMDB0000939	− 1.94	0.26	1.04E-04	1.80
Serotonin	HMDB0000259	− 3.19	0.11	1.21E-02	1.27
Cytidine 5′-diphosphocholine (CDP-choline)	HMDB0001413	− 3.90	0.07	5.29E-03	1.74
Adenosine	HMDB0000050	− 4.70	0.04	6.09E-05	1.57
Glyceric acid		− 5.07	0.03	7.26E-12	1.78
Adenosine monophosphate (AMP)	HMDB0000045	− 7.32	0.01	9.07E-09	1.77
Guanosine-5′-monophosphate (GMP)	HMDB0001397	− 10.60	0.0006	1.11E-04	1.88
5′-Inosinic acid (IMP)		− 10.99	0.0005	3.15E-05	1.62

### Pathway enrichment analysis and pathway ranking

**Fig. 3:**
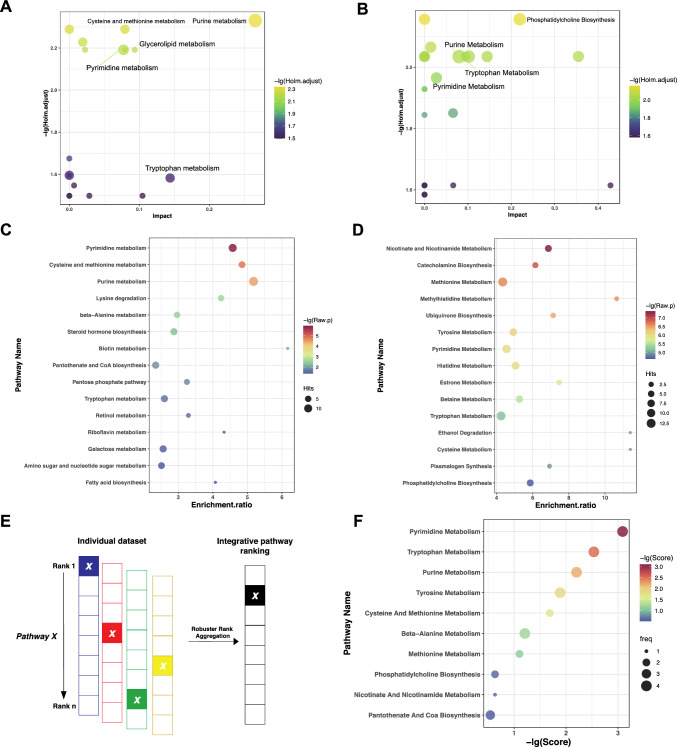
Prioritization of pathways in 3 × Tg-AD mice by order statistics. **a** Enrichment analysis in KEGG. **b** Enrichment analysis in SMPDB. **c** MSEA in KEGG. **d** MSEA in SMPDB. **e** Strategy for ranking pathways. **f** The ranking list by combing 4 database analyses

KEGG pathway analysis showed that these DEMs were primarily and significantly enriched in purine metabolism, cysteine and methionine metabolism, glycerophospholipid metabolism, and tryptophan metabolism (Fig. 3a). SMPDB analysis revealed that more pathways were significantly enriched, including phosphatidylcholine biosynthesis, tyrosine metabolism, betaine metabolism, purine metabolism, and tryptophan pathways (Fig. 3b). To obtain a broad overview of metabolite alterations, we performed MSEA analysis of 256 metabolites in KEGG and SMPDB, respectively. The results showed that the top 3 significantly enriched pathways in KEGG were pyrimidine metabolism, cysteine and methionine metabolism, and purine metabolism (Fig. 3c). In the SMPDB, nicotinate and nicotinamide metabolism, catecholamine biosynthesis, and methionine metabolism ranked in the top 3 (Fig. 3d). Combined with above significant pathway (*p* < 0.05 and impact > 0) to rank important pathways using order statistics, the results highlighted that pyrimidine metabolism, tryptophan metabolism, and purine metabolism were the priority pathways (Fig. 3e, f, Fig. S3 and Table S5). The DEMs associated with these pathways are listed in Table 2.

**Table 2:** Major metabolic pathways associated with differential expression metabolites

No	Metabolic pathway	Differential expression metabolites
1	Pyrimidine metabolism^a^	CMP, 3-Ureidopropionate ((3-Ureidopropionc acid), Thymidine
2	Tryptophan metabolism^a^	N-Acetylserotonin, Serotonin
3	Purine metabolism^a^	AMP, IMP, GMP, Adenosine, Xanthosine, Uric acid
4	Tyrosine metabolism^a^	3,4-Dihydroxymandelate
5	Cysteine and methionine metabolism^a^	S-Adenosyl-l-methionine (SAM), S-Adenosyl-l-homocysteine (SAH)
6	Glycerophospholipid metabolism	CDP-Choline, Acetylcholine
7	Glycerolipid metabolism	Glyceric acid
8	Pantothenate and CoA biosynthesis	3-Ureidopropionate (3-Ureidopropionc acid)
9	beta-Alanine metabolism	3-Ureidopropionate (3-Ureidopropionc acid)
10	Glyoxylate and dicarboxylate metabolism	Glyceric acid

^a^Indicated the pathway is significant in robust rank aggregation

### The interaction of differential metabolites from purine metabolism

**Fig. 4:**
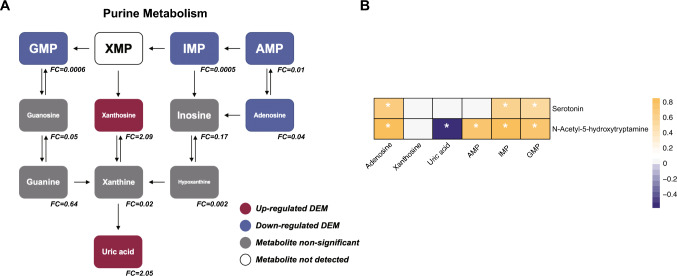
The differential metabolites in purine metabolic pathway and the correlation between metabolites. **a** The differential metabolites involved in purine metabolic pathway. **b** Correlation analysis of DEMs in purine and tryptophan metabolism. The white asterisk (*) indicates significant difference (*p* < 0.05), *FC* foldchange

In our results, a majority of DEMs were mainly enriched in purine metabolism pathway. The DEMs in purinergic metabolites were associated with other signaling molecules to form a complex landscape, including adenosine monophosphate (AMP), 5′-Inosinic acid (IMP), adenosine, guanosine-5′-monophosphate (GMP), uric acid, and xanthosine (Fig. 4a). In correlation analysis, we found that there was significant correlation between DEMs in purine metabolism and DEMs in tryptophan metabolism. Serotonin was significantly positively correlated with adenosine, IMP and GMP. In addition, N-acetyl-5-hydroxytryptamine was significantly positively correlated with adenosine, AMP, IMP and GMP and negatively correlated with uric acid (Fig. 4b).

### Important metabolic pathways and abundance of important differential metabolites between AD and control groups

**Fig. 5:**
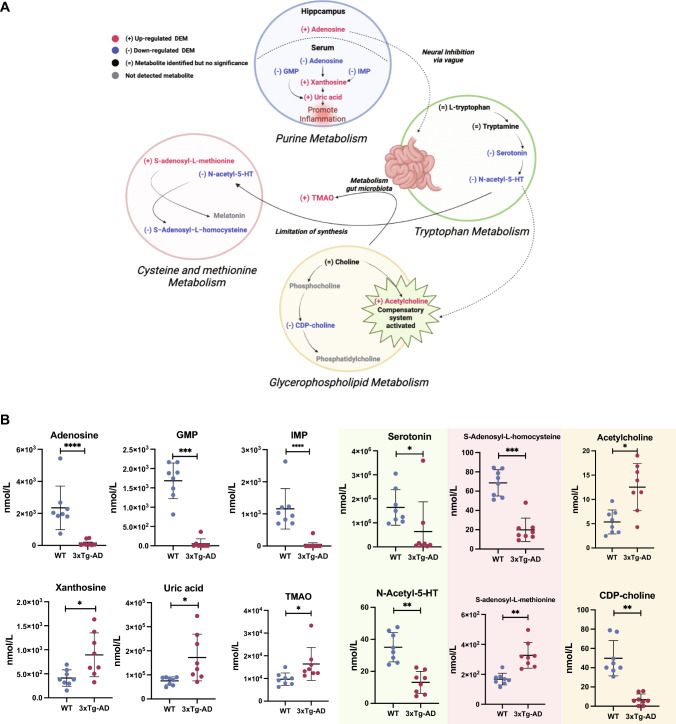
Summary of several important metabolic pathways. **a** The reactions within each metabolite are indicated by black arrows. The up-regulated DEMs in pathway are shown in red font. The down-regulated DEMs in pathway are shown in blue font. Metabolites whose concentrations were measured but did not differ are indicated by black text. The gray text indicates the metabolite is not to be detected in serum (Created with BioRender.com.) **b** Quantification values of DEMs involved in important pathways between 3 × Tg AD and WT mice. The blue background indicates DEMs from purine metabolism. The green background indicates DEMs from tryptophan metabolism. The red background indicates DEMs from cysteine and methionine metabolism. The yellow background indicates DEMs from glycerophospholipid metabolism. *p < 0.05, ** *p* < 0.01, *** p < 0.001, **** p < 0.0001, Limma moderated *t-test*

Several important pathways including purine, tryptophan, cysteine and methionine, and glycerophospholipid metabolic pathways and their possible interactions are outlined in Fig. 5a. The concentrations of the 12 important serum differential metabolites between AD and control are shown in Fig. 5b. They were involved in purine metabolism, tryptophan metabolism, cysteine and methionine metabolism, and glycerophospholipid metabolism. Among these, the levels of GMP, adenosine, IMP, serotonin, and CDP-choline were decreased, while acetylcholine, xanthosine, uric acid, trimethylamine-N-oxide (TMAO), N-Acetyl-5-hydroxytryptamine (N-acetyl-5-HT), S-adenosyl-l-methionine (SAM), and S-Adenosyl-l-homocysteine (SAH) were increased in the serum of AD mice.

## Discussion

In this study, a targeted metabolomics approach was used to study the changes of metabolites in the serum of 6-month-old 3 × Tg-AD mice. The profiles of these target metabolites showed a significant separation trend between the 3 × Tg-AD and WT mice groups and was able to distinguish the two groups. A total of 49 DEMs were identified between the two groups. Those DEMs were mainly involved in pyrimidine, purine, tryptophan and cysteine and methionine metabolism.

Disturbances in pyrimidine metabolism have recently been frequently associated with AD (Muguruma et al. 2020). In this study, the serum levels of 3-ureidopropionate and thymidine in AD mice were increased, while the levels of cytidine 5′-monophosphate (CMP) were decreased. The increase of 3-ureidopropionate suggests that the degradation of pyrimidine is accelerated (Southern et al. 2015). It acts as an endogenous neurotoxin by inhibiting mitochondrial energy metabolism, leading to secondary, energy-dependent excitotoxicity (Kölker et al. 2001). 3-ureidopropionate has been reported as one of the important factors in AD prediction (Rule et al. 2004).

Neurodegenerative disorders like AD can be attributed to abnormal purinergic signal spread (Huang et al. 2021; Woods et al. 2016). In the present study, upstream DEMs such as adenosine, AMP, IMP and GMP had low concentrations, while downstream DEMs (uric acid and xanthosine) had significantly increased concentrations in the serum of AD mice. Adenosine is an endogenous autacoid with a wide range of roles of inhibitory neuromodulator (Alonso-Andres et al. 2018; Snyder 1985; Merighi et al. 2022; Silva et al. 2018; Liu et al. 2019b). Similarly, its levels in blood were found to be decreased with advancing age and were significantly decreased in an aging and AD mice model (Sanchez-Melgar et al. 2020; Simard et al. 2019). Another recent study showed that it was down-regulated in the hippocampus of AD model mice and increased after drug treatment (Liu et al. 2019a). However, other studies have observed that its levels are increased in the blood of AD patients (Teruya et al. 2021; González-Domínguez et al. 2015) and in the hippocampus of 3 × Tg-AD mice (Zhao et al. 2021b). These discrepancies could potentially arise from the development of AD. Adenosine levels was impacted by serum adenosine deaminase which the activity was changed with aging (Vasudha et al. 2006; Sánchez-Melgar et al. 2020). Additionally, the colocalization of A1 receptors (A1R) with Aβ in senile plaques was observed in post-mortem hippocampal tissue of AD patients and the activity of A1R can meditated tau phosphorylation and translocation (Angulo et al. 2003). Adenosine is a neuroprotective agent that binds to adenosine receptors on cell membranes (particularly A1and A2A adenosine receptors) (Chang et al. 2021). Adenosine and its receptors are potential targets for AD (Liu et al. 2019a). We speculated that adenosine in periphery is associated with the increase of receptor in brain tissue. However, the strength of these links needs further exploration. Uric acid is an endogenous antioxidant and the main end product of purine metabolism (Tan et al. 2016). Some studies have shown that it was down-regulated in the blood of AD patients and associated with poorer cognitive function and an increased risk of MCI or overall dementia (Kim et al. 2020; Mullan et al. 2018; Liu et al. 2017; Euser et al. 2009). However, previous studies have demonstrated that Aβ expressions lead to a shift in purine metabolism, resulting in an increase of uric acid in AD mice (Esteve et al. 2017;Li et al. 2016; Munasinghe et al. 2022). Uric acid may also be associated with increased neuroinflammation in AD (Esteve et al. 2017). One hypothesis proposed that in some cases, particularly during the early stages of disease, although uric acid causes inflammation, it may be increased by antioxidant defenses against oxidation and free radicals (Ames et al. 1981; Bowman et al. 2010; Du et al. 2016). Therefore, AD may be underlined by low-grade chronic inflammation, both at local and peripheral level (Guzman-Martinez et al. 2019). Clearly, this hypothesis needs to be further confirmed.

Moreover, while serotonin is widely accepted to be related to depression, disruption of serotonergic signaling has been shown to be strongly associated with the pathogenesis of AD (Whiley et al. 2021; Smith et al. 2017). In this study, serotonin and N − acetyl − 5 − hydroxytryptamine (N-acetyl-5-HT), which are involved in tryptophan metabolism, were down-regulated in the serum of AD mice. The decrease of serotonin in serum and CSF has been observed, and its receptors were also found decrease in post-mortem brains of AD patient (Whiley et al. 2021; Tohgi et al. 1992; Garcia-Alloza et al. 2005). N-acetyl-5-HT is an intermediate substance between serotonin and melatonin. N-acetyl-5-HT and melatonin showed neuroprotection against amyloid-β peptide aggregation and cytotoxicity (Hornedo-Ortega et al. 2018; Sun et al. 2022; Oxenkrug 2005). Compelling evidence showed that a serotonin degeneration and was associated with an increase in the Aβ-related inflammatory response (Liu et al. 2008; Metaxas et al. 2019). The inflammatory process in AD is characterized by shunted tryptophan metabolism away from serotonin (Willette et al. 2021). The initial degeneration of serotonin may trigger a spread of AD pathology and accumulating studies suggested that serotonin is a potential early hallmarks of AD pathology (Gallo et al. 2021). Together, Aβ deposit may triggered an inflammation with the presence of dysregulated tryptophan metabolism.

Cysteine and methionine metabolic pathway was also a pathway of interest, and SAH and SAM were involved. SAH were decreased, while SAM was increased in the serum of AD mice. They are important precursors in melatonin synthesis. A growing body of research suggests that sleep deprivation is considered as a risk factor for AD. Supplementation of melatonin bring beneficial effect to AD mice and patients (Uddin et al. 2020; Rudnitskaya et al. 2015; Irwin and Vitiello 2019). N-acetyl-5-HT is converted to melatonin by O-methylation of the 5-hydroxy group using S-adenosyl-l-methionine as the methyl donor under the catalysis of hydroxyindole-O-methyltransferase (Itoh et al. 1997). Therefore, the deficiency of N-acetyl-5-HT may reduce the synthesis of melatonin and SAH and contributed to accumulate SAM. Methionine has been demonstrated to alleviate a variety of risk factors and hallmarks associated with AD (Shea and Chan 2008). Methionine has been found to be down-regulated in the hippocampus of 3 × Tg-AD mice in our previous study (Zhao et al. 2021b). An abnormal SAM/SAH ratio is decreased as a character of cognitive decline and death with dementia in elderly populations (Linnebank et al. 2010; Mihara et al. 2022; Guiraud et al. 2017; Mihara et al. 2022). SAM and SAH have been observed to be increased in the brain of AD patients (Mahajan et al. 2020). Another study reported that lower SAH and higher SAM concentrations in the plasma of AD patients (Guiraud et al. 2017). Besides, a previous study proposed that a metabolic link between methionine and phospholipid metabolism may contribute to cerebrovascular and neurodegenerative changes in AD (Obeid and Herrmann 2006).

It is widely known that dysregulation of cholinergic and monoaminergic systems is associated with the pathogenesis of AD (Zong et al. 2022). Acetylcholinesterase (AChE) inhibitor currently is used in AD therapy (Bortolami et al. 2021). In this study, the high levels of acetylcholine were detected in the serum of AD mice. One study observed that acetylcholine was increased in the temporal cortex of AD patients (Kim et al. 2019). On the contrary, lower levels of acetylcholine were observed in plasma of mild cognitive impairment (MCI) (Peña-Bautista et al. 2020), CSF of AD patients (Kumar et al. 1989) and the hippocampus of AD patients (Liu et al. 2021). The dysregulated AChE has a significant impact on availability of acetylcholine (Sadia et al. 2023) and AChE activity progressively diminishes as the severity of dementia advances (Lane et al. 2006). In addition, a previous study speculated that a compensatory system of acetylcholine was activated to reduce Aβ accumulation when the level of serotonin was abnormal in AD (Madsen et al. 2011). Therefore, the compensatory reaction and AChE activity may contribute to the high level of acetylcholine in serum. Another cholinergic precursor, CDP-choline was found to decrease in the serum of 3 × Tg-AD mice. CDP-choline has been shown to elicit advantageous effects in the brain including inhibition of apoptosis, enhancement of neuroplasticity and synthesis of phospholipids and acetylcholine (Gareri et al. 2017; Adibhatla et al. 2002; Fioravanti and Yanagi 2000, 2004).

Furthermore, we identified up-regulation of TMAO levels in 3xTg-AD mice serum. TMAO is a metabolite derived from choline in intestinal flora and have been considered as a risk factor of neurological disorders (Chhibber-Goel et al. 2017; Arrona Cardoza et al. 2022; Buawangpong et al. 2022; Janeiro et al. 2018). The higher levels of TMAO were commonly observed in the plasma, CSF, or brain in both humans or mice (Li et al. 2018; Chen et al. 2022; Vogt et al. 2018; Govindarajulu et al. 2020). Previous studies have shown that peripheral TMAO levels are highly correlated with levels in the brain (Brunt et al. 2021). Elevated serum concentrations of TMAO increased the cellular inflammatory response probably by activating microglia and astrocytes and its perfusion of the brain parenchyma caused progression of small-vessel diseases leading to white matter degeneration observed in AD (Buawangpong et al. 2022; Pietroboni et al. 2020; Brown et al. 2002). These findings support that the disturbance of intestinal flora, along with its metabolites, can reaches central nervous system (CNS), and may, therefore, impairs neurological function (Cryan et al. 2020; Rutsch et al. 2020). Moreover, in this study, serum levels of glyceric acid were reduced in AD mice. Similarly, glyceric acid was down-regulated in the hippocampus of APP/PS1 (AD) mice (Hunsberger et al. 2020) and 3 × Tg-AD mice (Zhao et al. 2021b).

We note that the DEMs identified in this study may interact with one another or converge on a shared mechanism. Adenosine are distributed both presynaptically and postsynaptically in brain leading to inhibit the releases of serotonin and other excitatory neurotransmitter (van Calker et al. 2019; Ribeiro 1995). Here, DEMs from purine pathway (adenosine, AMP, GMP and IMP) and tryptophan pathways (serotonin and N-acetyl-5-HT) suggests showed strong correlation. A novel perspective suggests that gastrointestinal physiology is highly regulated by innervation from the CNS via vagus nerve (Sundman et al. 2017; Mercado et al. 2022; Aaldijk and Vermeiren 2022; El-Merahbi et al. 2015). A study suggested that the accumulation or degradation of adenosine both regulate 5-hydroxytryptamine release from a human enterochromaffin cell through different adenosine receptors (Christofi et al. 2004). The gastrointestinal tract is largest serotonin pool (Yano et al. 2015). Thus, the decline in peripheral serotonin and N-acetyl-5-HT may be caused by dysregulation of neurotransmitters like adenosine. In addition, as mentioned above, serotonin and TMAO are associated with the gut microbiota (Yano et al. 2015; Brunt et al. 2021), supporting that the disturbance of intestinal flora and its metabolites may be associated with the pathogenesis of AD (Cryan et al. 2020; Rutsch et al. 2020). Moreover, compared with the previous study in the hippocampus of 3 × Tg-AD mice (Zhao et al. 2021b), the expression trend of 6 DEMs (3-Ureidopropionate, CMP, adenosine, AMP, IMP and CDP-choline) was reversed. Although the reasons require further investigation, the results suggest that purine and pyrimidine metabolism may play a key role in the pathogenesis of AD.

It is important to note that although we did our best to compare the DEMs and their associated pathways screened in this study with previous studies, their specificity, that is whether they are AD-specific or may represent a broader range of changes associated with aging or different neurodegenerative conditions, needs to be validated by experiments or datasets that include additional animal or human samples, especially human samples.

## Conclusion

In the present study, we have carried out a serum-targeted metabolomics study in 6-month-old 3 × Tg-AD mice versus controls and observed broad dysregulation of metabolites. Of these, adenosine may play a role in purine metabolism and interacts with neuromodulator networks. The decrease in serotonin, and alterations in N-acetyl-5-HT and SAM involved in the synthesis of melatonin, suggest a relationship between depression, sleep disorders and AD. Acetylcholine and CDP-choline may act as a compensation to reduce the contraction of nervous system. TMAO and serotonin suggest a link between AD and gut microbiota for metabolites in the serum. A dynamic bidirectional communication system between the gut, microbiome, the blood, and CNS may play a role in AD. Here, the alteration of these metabolic pathways in blood seem to converge on promoting inflammation and were associated with gut microbiota. This study will provide new insights into the therapeutic and diagnostic markers of AD. However, further studies and validation in animals and populations are required.

## Supplementary Information

Below is the link to the electronic supplementary material.

Supplementary file1 (EPS 681 KB)

Supplementary file2 (EPS 2156 KB)

Supplementary file3 (EPS 781 KB)

Supplementary file4 (XLSX 11 KB)

Supplementary file5 (XLSX 36 KB)

Supplementary file6 (XLSX 18 KB)

Supplementary file7 (XLSX 11 KB)

Supplementary file8 (XLSX 12 KB)

## Data Availability

All raw data and corresponding parameter have been deposited as online resource to the Figshare database with the name: “Investigating Metabolic Dysregulation in Serum of Triple Transgenic Alzheimer’s Disease Male Mice: Implications for Pathogenesis and Potential Biomarkers”. (10.6084/m9.figshare.22731110).
